# Risk of New-Onset Type 2 Diabetes Among Vaccinated Adults After Omicron or Delta Variant SARS-CoV-2 Infection

**DOI:** 10.1001/jamanetworkopen.2025.2959

**Published:** 2025-04-02

**Authors:** Liang En Wee, Jue Tao Lim, Enoch Xueheng Loy, Calvin J. Chiew, E. Shyong Tai, Su Chi Lim, Yong Mong Bee, Sock Hwee Tan, Charmaine Qing Fei Chan, Wai Leng Chow, James Wei Luen Yip, Khung Keong Yeo, Benjamin Ong, David Chien Boon Lye, Mark Yan Yee Chan, Derek J. Hausenloy, Kelvin Bryan Tan

**Affiliations:** 1 National Centre for Infectious Diseases, Singapore; 2 Duke-NUS Medical School, National University of Singapore, Singapore; 3 Department of Infectious Diseases, Singapore General Hospital, Singapore; 4 Lee Kong Chian School of Medicine, Nanyang Technological University, Singapore; 5 Ministry of Health, Singapore; 6 Yong Loo Lin School of Medicine, National University of Singapore, Singapore; 7 Saw Swee Hock School of Public Health, National University of Singapore, Singapore; 8 Department of Medicine, Yong Loo Lin School of Medicine, Singapore; 9 Clinical Research Unit, Khoo Teck Puat Hospital, Singapore; 10 Diabetes Centre, Admiralty Medical Centre, Singapore; 11 Department of Endocrinology, Singapore General Hospital, Singapore; 12 Department of Cardiology, National University Heart Centre, Singapore; 13 Department of Cardiology, National Heart Centre Singapore, Singapore; 14 Department of Infectious Diseases, Tan Tock Seng Hospital, Singapore; 15 National Heart Research Institute Singapore, National Heart Centre, Singapore; 16 The Hatter Cardiovascular Institute, University College London, London, United Kingdom

## Abstract

This cohort study estimates the risk of new-onset type 2 diabetes after Delta or Omicron variant SARS-CoV-2 infection among vaccinated adults in Singapore.

## Introduction

New-onset diabetes after SARS-CoV-2 infection has been documented.^1,2,3,4^ Milder Omicron variant infection and booster vaccination may mitigate risk, but most available studies predate Omicron emergence and booster rollout.^1,2,3^ We estimated risk of new-onset type 2 diabetes (T2D) after SARS-CoV-2 infection among boosted adults during Delta- and Omicron-predominant transmission in Singapore.

## Methods

This cohort study used Singapore’s national COVID-19 registry to construct cohorts with a positive or negative SARS-CoV-2 test result between September 1, 2021, and December 31, 2022. Vaccination status and new-onset T2D were determined using the National Immunization Registry^5^ and the National Diabetes Database (eMethods 1 in Supplement 1). Covariates included demographics (age, sex, ethnicity, socioeconomic status), vaccination status at test (index) date (T_0_), and comorbidities. Individuals were excluded for death within 30 days of T_0_, missing sociodemographic data, SARS-CoV-2 infection within 300 days of T_0_, or preexisting T2D (eFigure in Supplement 1). The primary outcome of interest was new-onset T2D 31 to 300 days after T_0_ among individuals with vs without SARS-CoV-2 infection. This study was considered national public health research under the Singapore Infectious Diseases Act; thus, patient consent and institutional review board approval were not required. Reporting followed the STROBE guideline.

Baseline characteristics and between-group standardized mean differences (SMDs) were computed; overlap weighting was used to adjust for differences. Risk of new-onset T2D was estimated using competing-risks regression with death as a competing risk, with overlap weights applied; subgroup analyses included ethnicity, COVID-19 vaccination, and infection severity. Sensitivity analyses were conducted with inverse probability weights or the doubly robust approach and the negative-outcome control (solid-organ malignant neoplasm). We constructed a historical cohort for influenza hospitalizations separately; risk of new-onset T2D after hospitalization for COVID-19 vs influenza was contrasted using competing-risks regression (eMethods 2 in Supplement 1). Analyses were conducted using R, version 4.3.1 (R Project for Statistical Computing). 95% CIs excluding 1 indicated significance.

## Results

We compared individuals with vs without SARS-CoV-2 infection during Delta (82 212 vs 531 855) and Omicron (972 610 vs 1 039 276) predominance. SMDs were below 0.05 after weighting (Table 1). No overall increased risk of new-onset T2D was observed during Delta (hazard ratio [HR], 0.99 [95% CI, 0.92-1.06]) or Omicron (HR, 1.00 [95% CI, 0.97-1.03]) predominance; sensitivity analyses did not notably alter these estimates (Table 2). In subgroup analyses, increased postinfectious risk of T2D was observed during Delta predominance among Indian individuals but not among Chinese or Malay individuals. During Omicron predominance, elevated postinfectious risk of T2D (HR, 1.50 [95% CI, 1.06-2.11]) was observed among unvaccinated or partially vaccinated individuals but not among fully vaccinated or boosted individuals. T2D risk was not elevated among patients with mild SARS-CoV-2 infection, but those hospitalized with COVID-19 had increased postinfectious risk. Elevated postinfectious risk persisted in hospitalized patients with COVID-19 during Omicron predominance, even after excluding steroid-treated individuals. Compared with historical influenza hospitalizations, patients with COVID-19 had 45.0% higher postacute risk of T2D during Delta predominance vs 56.0% higher risk during Omicron predominance. For the negative-outcome control (malignant neoplasm), between-group risk did not differ notably.

**Table 1.** Baseline Characteristics of Individuals With vs Without SARS-CoV-2 Infection During Delta- and Omicron-Predominant Transmission

Characteristic	Delta-predominant transmission				Omicron-predominant transmission			
	No. (%) of patients		SMD		No. (%) of patients		SMD	
	With infection (n = 82 212)	Without infection (n = 531 855)	Baseline	After weighting^a^	With infection (n = 972 610)	Without infection (n = 1 039 276)	Baseline	After weighting^a^
Age distribution, y								
18-29	15 632 (19.0)	110 124 (20.7)	0.04	0	228 924 (23.5)	232 780 (22.4)	0.03	0
30-39	15 208 (18.5)	114 887 (21.6)	0.08	0	223 405 (23.0)	224 040 (21.6)	0.03	0
40-49	16 536 (20.1)	108 173 (20.3)	0.01	0	198 503 (20.4)	205 699 (19.8)	0.02	0
50-59	17 137 (20.8)	91 215 (17.2)	0.09	0	153 488 (15.8)	174 478 (16.8)	0.03	0
60-69	11 199 (13.6)	66 602 (12.5)	0.03	0	106 237 (10.9)	127 153 (12.2)	0.04	0
70-79	4540 (5.5)	29 907 (5.6)	0	0	44 313 (4.6)	55 920 (5.4)	0.04	0
≥80	1960 (2.4)	10 947 (2.1)	0.02	0	17 740 (1.8)	19 206 (1.8)	0	0
Sex								
Female	35 707 (43.4)	281 390 (52.9)	0.19	0	511 747 (52.6)	540 618 (52.0)	0.01	0
Male	46 505 (56.6)	250 465 (47.1)	0.19	0	460 863 (47.4)	498 658 (48.0)	0.01	0
Ethnicity								
Chinese	56 317 (68.5)	392 241 (73.7)	0.12	0	742 482 (76.3)	781 208 (75.2)	0.03	0
Indian	8592 (10.5)	52 756 (9.9)	0.02	0	68 949 (7.1)	96 885 (9.3)	0.08	0
Malay	14 824 (18.0)	66 656 (12.5)	0.15	0	131 311 (13.5)	119 433 (11.5)	0.06	0
Other^b^	2479 (3.0)	20 202 (3.8)	0.04	0	29 868 (3.1)	41 750 (4.0)	0.05	0
Housing type								
Public, No. of rooms								
1-2	6036 (7.3)	20 945 (3.9)	0.15	0	34 398 (3.5)	39 787 (3.8)	0.02	0
3	16 460 (20.0)	74 767 (14.1)	0.16	0	133 034 (13.7)	139 554 (13.4)	0.01	0
4	30 916 (37.6)	172 325 (32.4)	0.11	0	339 294 (34.9)	323 214 (31.1)	0.08	0
5	25 423 (30.9)	223 622 (42.0)	0.23	0	409 126 (42.1)	448 886 (43.2)	0.02	0
Private	3377 (4.1)	40 196 (7.6)	0.15	0	56 758 (5.8)	87 835 (8.5)	0.10	0
Comorbidity burden (CCI score)^c^								
None (0)	75 694 (92.1)	498 536 (93.7)	0.06	0	907 613 (93.3)	975 800 (93.9)	0.02	0
Mild (1-2)	5400 (6.6)	29 161 (5.5)	0.05	0	55 147 (5.7)	54 955 (5.3)	0.02	0
Moderate (3-4)	678 (0.8)	2459 (0.5)	0.05	0	5755 (0.6)	4970 (0.5)	0.02	0
Severe (≥5)	440 (0.5)	1699 (0.3)	0.03	0	4095 (0.4)	3551 (0.3)	0.01	0
Vaccination status at T_0_^d^								
Unvaccinated or partially vaccinated	3850 (4.7)	21 392 (4.0)	0.03	0	8488 (0.9)	23 858 (2.3)	0.11	0
Fully vaccinated	72 792 (88.5)	452 145 (85.0)	0.10	0	94 923 (9.8)	128 093 (12.3)	0.08	0
Boosted	5570 (6.8)	58 312 (11.0)	0.15	0	837 709 (86.1)	827 600 (79.6)	0.17	0
Doubly boosted	0	6 (0.001)	0	0	31 490 (3.2)	59 725 (5.7)	0.12	0

Abbreviations: CCI, Charleson Comorbidity Index; SMD, standardized mean difference; T_0_, index (test) date.

^a^ Calculated after overlap weighting. An SMD less than 0.1 between groups with vs without SARS-CoV-2 infection after weighting was taken as the threshold for good covariate balance.

^b^ Includes individuals of other ethnicity (eg, Eurasian, Arab) or multiple ethnicities.

^c^ Defined using the CCI, consisting of the following: myocardial infarction, chronic heart failure, peripheral vascular disease, cerebrovascular accident, dementia, chronic obstructive pulmonary disease, connective tissue disease, peptic ulcer disease, diabetes, hemiplegia, liver disease, moderate to severe kidney impairment, solid tumor, leukemia, HIV, or AIDS.

^d^ Defined as did not complete primary vaccination series (ie, either unvaccinated or partially vaccinated with a single dose of mRNA COVID-19 vaccine, either BNT162b2 or mRNA-1273), completed primary vaccination series only (having completed a primary vaccine series of 2 doses of mRNA COVID-19 vaccine, at least 8 weeks apart), boosted, or doubly boosted (booster dose defined as an additional mRNA vaccine dose 6-9 months after the second dose). During Delta-predominant transmission, fourth vaccine doses had yet to be rolled out as part of the national vaccination program.

**Table 2.** Risk and Excess Burden of New-Incident T2D After SARS-CoV-2 Infection Among Patient and Control Groups During Delta- and Omicron-Predominant Transmission

Characteristic	New-onset T2D among patients with vs without SARS-CoV-2 infection		No./total No. (%) of patients with new-onset T2D	
	AHR (95% CI)	Excess burden, weighted, per 1000 persons, (95% CI)	With SARS-CoV-2 infection	Without SARS-CoV-2 infection
**Delta-predominant transmission**				
Entire cohort with vs without SARS-CoV-2 infection^a^	0.99 (0.92-1.06)	−0.15 (−0.92 to 0.63)	948/82 212 (1.2)	5378/531 855 (1.0)
Sensitivity analysis				
Inverse propensity weighted and regression adjusted^b^	0.97 (0.90-1.04)	−0.36 (−1.09 to 0.37)	948/82 212 (1.2)	5378/531 855 (1.0)
Inverse probability weighted, together with covariate adjustment^c^	0.96 (0.90-1.04)	−0.36 (−1.09 to 0.37)	948/82 212 (1.2)	5378/531 855 (1.0)
Subgroup^a^				
Ethnicity^d^				
Chinese	0.96 (0.88-1.04)	−0.50 (−1.41 to 0.41)	618/56 317 (1.10)	3851/392 241 (0.98)
Malay	0.91 (0.77-1.08)	−1.05 (−2.94 to 0.84)	169/14 824 (1.14)	775/66 656 (1.16)
Indian	1.29 (1.07-1.55)	3.63 (0.78-6.47)	144/8592 (1.68)	612/52 756 (1.16)
Vaccination status				
Unvaccinated or partially vaccinated	1.30 (0.98-1.73)	3.68 (−0.57 to 7.94)	67/3850 (1.74)	194/21 392 (0.91)
Fully vaccinated	1.01 (0.93-1.09)	0.07 (−0.73 to 0.87)	787/72 792 (1.08)	4040/452 145 (0.89)
Boosted	0.77 (0.62-0.95)	−4.95 (−8.51 to −1.39)	94/5570 (1.69)	1144/58 312 (1.96)
Severity of initial infection				
Mild, not requiring initial hospitalization	0.95 (0.89-1.03)	−0.50 (−1.27 to 0.27)	813/76 982 (1.07)	5378/531 855 (1.01)
Hospitalized with COVID-19	1.32 (1.10-1.59)	5.99 (1.74-10.24)	135/5230 (2.58)	5378/531 855 (1.01)
Hospitalized with acute COVID-19 and not treated with steroids	1.14 (0.92-1.40)	2.45 (−1.76 to 6.67)	97/4502 (2.15)	5378/531 855 (1.01)
COVID-19 hospitalizations vs historical influenza hospitalizations^e^	1.45 (1.02-2.09)	6.89 (1.09-12.69)	135/5230 (2.58)	42/3304 (1.27)
**Omicron-predominant transmission**				
Entire cohort with vs without SARS-CoV-2 infection^a^	1.00 (0.97-1.03)	−0.04 (−0.30 to 0.22)	8385/972 610 (0.9)	9349/1 039 276 (0.9)
Sensitivity analysis				
Inverse propensity weighted and regression adjusted^b^	1.00 (0.97-1.03)	−0.01 (−0.27 to 0.25)	8385/972 610 (0.9)	9349/1 039 276 (0.9)
Inverse probability weighted, together with covariate adjustment^c^	1.00 (0.97-1.03)	−0.01 (−0.27 to 0.25)	8385/972 610 (0.9)	9349/1 039 276 (0.9)
Subgroup^a^				
Ethnicity^d^				
Chinese	0.99 (0.96-1.02)	0.99 (0.96-1.02)	6036/742 482 (0.81)	6708/781 208 (0.86)
Malay	0.97 (0.90-1.04)	0.97 (0.90-1.04)	1382/131 311 (1.05)	1367/119 433 (1.14)
Indian	1.04 (0.94-1.14)	1.04 (0.94-1.14)	776/68 949 (1.13)	1038/96 885 (1.07)
Vaccination status				
Unvaccinated or partially vaccinated	1.50 (1.06-2.11)	2.16 (0.25-4.06)	63/8488 (0.74)	81/23 858 (0.34)
Fully vaccinated	1.05 (0.94-1.17)	0.28 (−0.37 to 0.92)	589/94 923 (0.62)	719/128 093 (0.56)
Boosted	0.99 (0.95-1.02)	−0.13 (−0.41 to 0.15)	7017/837 709 (0.84)	7276/827 600 (0.88)
Doubly boosted	1.02 (0.93-1.12)	0.46 (−1.56 to 2.48)	716/31 490 (2.27)	1273/59 725 (2.13)
Severity of initial infection				
Mild, not requiring initial hospitalization	0.99 (0.96-1.02)	−0.12 (−0.38 to 0.14)	8064/961 186 (0.84)	9349/1 039 276 (0.90)
Hospitalized for COVID-19	1.29 (1.15-1.46)	6.13 (3.11-9.14)	321/11 424 (2.81)	9349/1 039 276 (0.90)
Hospitalized for acute COVID-19 and not treated with steroids	1.21 (1.06-1.38)	4.34 (1.26-7.41)	262/10 130 (2.59)	9349/1 039 276 (0.90)
COVID-19 hospitalizations vs historical influenza hospitalizations^e^	1.56 (1.12-2.19)	7.86 (3.01-12.70)	321/11 424 (2.81)	42/3304 (1.27)

Abbreviations: AHR, adjusted hazard ratio; T2D, type 2 diabetes.

^a^ Overlap weighted and regression adjusted based on demographic characteristics (age, sex, and ethnicity), socioeconomic status (housing type), vaccination status (not fully vaccinated, fully vaccinated, or fully vaccinated and boosted), and comorbidities. The mean (SD) follow-up time for patients with vs without SARS-CoV-2 infection was 297.1 (22.9) and 297.8 (19.9) days during Delta predominance and 297.9 (19.3) vs 298.0 (19.1) days during Omicron predominance.

^b^ Inverse propensity weighted and regression adjusted based on demographic characteristics (age, sex, and ethnicity), socioeconomic status (housing type), vaccination status (not fully vaccinated, fully vaccinated, or fully vaccinated and boosted), and comorbidities.

^c^ Inverse probability weighted, together with covariate adjustment in the logistic regression step (doubly robust).

^d^ Individuals of other ethnicities were not analyzed as a separate category because of the relatively smaller number of individuals in this category.

^e^ Overlap weighted and regression adjusted based on demographic characteristics (age, sex, and ethnicity), socioeconomic status (housing type), vaccination status (vaccinated or unvaccinated for COVID-19 or influenza, respectively), comorbidities, and severity of initial hospitalization (needed intensive care unit, intensive care unit admission).

## Discussion

No overall increased risk of new-onset T2D after SARS-CoV-2 infection was observed in our cohort. Earlier pre-Omicron studies reported increased (27%-46%) diabetes risk after SARS-CoV-2 infection.^1,2,3^ Although increased risk was observed following Omicron infection in Hong Kong adults^4^ and no notable decrease was observed among boosted Korean individuals (vs primary vaccination),^6^ 58.4% of Hong Kong individuals had not completed primary vaccination and 66% of Korean individuals were boosted^4,6^; 89.4% of our population was boosted. Interethnic differences in insulin resistance pathophysiology may influence diabetogenic risk after infection.

Study limitations include results that may not be fully generalizable to populations of different ethnicities or lower vaccination uptake and absence of physical measurements. We observed no overall increased risk of new-onset T2D after mild SARS-CoV-2 infection among a highly vaccinated and boosted multiethnic Asian cohort during Omicron predominance; risk was elevated among Indian individuals during Delta predominance, unvaccinated individuals, and hospitalized patients with COVID-19. Risk of T2D after hospitalization was higher for the COVID-19 cohort vs the historical influenza cohort. Future research is required to evaluate the risk of new-onset T2D after COVID-19 vs other viral infections.

## Notes

Supplement 1. **eMethods 1.** National Diabetes Database: Data Sources and Definition of Outcome Measures (Type 2 Diabetes) **eMethods 2.** Additional Information on Statistical Methods and Sensitivity Analyses **eFigure.** Cohort Construction Flowchart
eReferences

Supplement 2.
Data Sharing Statement

## References

[1] Rezel-Potts E, Douiri A, Sun X, Chowienczyk PJ, Shah AM, Gulliford MC. Cardiometabolic outcomes up to 12 months after COVID-19 infection: a matched cohort study in the UK. PLoS Med. 2022;19(7):e1004052. doi:10.1371/journal.pmed.1004052 35853019 PMC9295991

[2] Xie Y, Al-Aly Z. Risks and burdens of incident diabetes in long COVID: a cohort study. Lancet Diabetes Endocrinol. 2022;10(5):311-321. doi:10.1016/S2213-8587(22)00044-4 35325624 PMC8937253

[3] Taylor K, Eastwood S, Walker V, ; Longitudinal Health and Wellbeing and Data and Connectivity UK COVID-19 National Core Studies; CONVALESCENCE study; OpenSAFELY collaborative. Incidence of diabetes after SARS-CoV-2 infection in England and the implications of COVID-19 vaccination: a retrospective cohort study of 16 million people. Lancet Diabetes Endocrinol. 2024;12(8):558-568. doi:10.1016/S2213-8587(24)00159-1 39054034 PMC7617111

[4] Xiong X, Lui DTW, Chung MSH, . Incidence of diabetes following COVID-19 vaccination and SARS-CoV-2 infection in Hong Kong: a population-based cohort study. PLoS Med. 2023;20(7):e1004274. doi:10.1371/journal.pmed.1004274 37486927 PMC10406181

[5] Wee LE, Pang D, Chiew C, . Long-term real-world protection afforded by third mRNA doses against symptomatic severe acute respiratory syndrome coronavirus 2 infections, coronavirus disease 19-related emergency attendances and hospitalizations amongst older Singaporeans during an Omicron XBB wave. Clin Infect Dis. 2023;77(8):1111-1119. doi:10.1093/cid/ciad345 37280047

[6] Huh K, Kim YE, Bae GH, . Vaccination and the risk of post-acute sequelae after COVID-19 in the Omicron-predominant period. Clin Microbiol Infect. 2024;30(5):666-673. doi:10.1016/j.cmi.2024.01.028 38331252

