# Supplementary material for: Risk of New-Onset Type 2 Diabetes Among Vaccinated Adults After Omicron or Delta Variant SARS-CoV-2 Infection
Source: JAMA Netw Open. 2025 Apr 2;8(4):e252959. doi: 10.1001/jamanetworkopen.2025.2959 (PMC11966305; doi:10.1001/jamanetworkopen.2025.2959)
Supplement: Supplement 1. — eMethods 1. National Diabetes Database: Data Sources and Definition of Outcome Measures (Type 2 Diabetes) eMethods 2. Additional Information on Statistical Methods and Sensitivity Analyses eFigure. Cohort Construction Flowchart eReferences [file jamanetwopen-e252959-s001.pdf]

Supplemental Online Content

Wee LE, Lim JT, Loy EX, et al. Risk of new-onset type 2 diabetes among vaccinated adults after Omicron or Delta variant SARS-CoV-2 infection. *JAMA Netw Open*. 2025;8(4):e252959. doi:10.1001/jamanetworkopen.2025.2959

eMethods 1. National Diabetes Database: Data Sources and Definition of Outcome Measures (Type 2 Diabetes)

eMethods 2. Additional Information on Statistical Methods and Sensitivity Analyses

eFigure. Cohort Construction Flowchart

eReferences

This supplemental material has been provided by the authors to give readers additional information about their work.

© 2025 Wee LE et al. *JAMA Network Open*.

eMethods 1. National Diabetes Database: Data Sources and Definition of Outcome Measures (Type 2 Diabetes)

The National Diabetes Database (NDD) is a nationwide registry for diabetes in Singapore, set up by the Ministry of Health (MOH), Singapore. The NDD unifies data from five data sources: **(1)** NEHR (the National Electronic Health Record), containing information on patient demographics, diagnoses, laboratory data and medication prescriptions**. (2,3)** EHINTS and EDW, which are cluster-wide hospital electronic medical records, containing information on referrals, billings, clinical procedures, diagnoses, laboratory data, medication prescriptions, and diabetic eye/foot screenings. **(4)** CHAS, the Community Health Assist Scheme, which allows Singaporean citizens to receive subsidies for medical care at private primary care (general practitioner) clinics and complements the Chronic Disease Management Programme (CDMP), the scheme that allows for usage of the national government-administered medical-savings scheme (Medisave) for outpatient treatment of chronic medical conditions, such as diabetes, at both public and private healthcare providers. **(5)** Omnibus, the administrative healthcare claims database, containing information on healthcare claims from both Medisave and Medishield, the national government-administered medical insurance scheme. Matching of the NDD with the national COVID-19 registry was performed by a trusted-third-party designated by the MOH through a secure anonymisation process and only anonymised data was transferred to the study team, as part of built-in safeguards to protect user privacy. Standard data cleaning procedures were carried out during cohort construction, including the exclusion of individuals with implausible ages (taken as age≥120 years in this case), implausible outcomes (eg. hospitalisation events occurring after death dates), and individuals with incomplete/missing sociodemographic data. However, as clinical information was derived from national databases maintained by the local MOH and linked back to death data obtained from the national registry of births/deaths, completeness of data was high and missing data was minimal. Individuals with missing data were excluded during the process of cohort construction; missing data was not imputed.

Type 2 diabetes was defined as either of the following: (1) a diagnosis of diabetes based on ICD-9/ICD-10 diagnosis codes in national healthcare claims data (ICD-9: at least one primary or secondary ICD-9 diagnosis code of 250.00, 250.02, 250.10, 250.12, 250.20, 250.22, 250.30, 250.32, 250.40, 250.42, 250.50, 250.52, 250.60, 250.62, 250.70, 250.72, 250.80, 250.82, 250.90, 250.92, 362.0x, 357.2 or 366.41; ICD-10: at least one primary or secondary ICD-10 diagnosis code starting with E11/E13). ICD-10-CM replaced the ICD-9-CM in 2012. (2) A positive laboratory test record (positive oral-glucose-tolerance-test≥11.0 mmol/L, fasting plasma glucose≥7.0 mmol/L, HbA1c≥7.0%) based on national guidelines. (3) A prescription record of diabetes medication for more than 30 days, including any of the following drug categories: biguanides, sulphonylureas, meglitinides, alpha-glucosidase inhibitors, thiazolidinediones, DPP-4 inhibitors, SGLT2 inhibitors, GLP-1 receptor agonists, and insulin preparations. As part of cohort construction, individuals with type-2 diabetes/prediabetes (where pre-diabetes was defined as a fasting plasma glucose 6.1-6.9 mmol/L, or a two-hour plasma glucose level of 7.8-11.0 mmol/L after oral-glucose-tolerance-test, based on national guidelines) recorded in the NDD at any time point before T_0 (test-date) were excluded.

© 2025 Wee LE et al. *JAMA Network Open*.

eMethods 2. Additional Information on Statistical Methods and Sensitivity Analyses

Baseline sociodemographic characteristics of the Delta/Omicron cohorts of infected cases and test-negative controls, along with standardised-mean-differences (SMDs) between groups, were computed. Logistic regression was used to estimate the propensity for enrolled individuals to belong to the infected group, taking sociodemographic characteristics and comorbidities as additional explanatory variables. Propensity scores were then estimated and overlap weights (to better account for outlying values and alleviate potential concerns regarding extreme weights generated using alternative weighting schemes) were computed as equal to the propensity score for test-negatives and 1-propensity score for test-positives.[1] Covariate balance was evaluated by comparing SMDs between unexposed and exposed groups, and an SMD < 0.1 was taken as the threshold for good balance.

Hazard-ratios (HRs) of new-onset type 2 diabetes were then estimated using hazard models with death taken as a competing risk, with overlap weights applied. Estimation of variance when weightings were applied was accomplished by using robust sandwich variance estimators. Excess burden (EB) per 1,000 individuals at 300 days of follow-up of new-onset type 2 diabetes and were computed based on differences in estimated incidence rates between infected cases and test-negative controls. Additional subgroup analyses were conducted by ethnicity (Chinese, Malay, Indian), severity of acute infection (non-hospitalised, hospitalised), and vaccination doses (0/1, 2, 3+). Risk trajectories were estimated between test positives versus negatives by employing the Kaplan-Meier approach, with the same overlap weights applied, to compare between-group differences in outcome probabilities over the post-acute follow-up period. Outcome probabilities were taken as 1-survival probabilities. Differences in probabilities of new-onset type 2 diabetes between test negatives and test-positives did not diverge significantly over the 31-300 day follow-up period.

We investigated the robustness of our results for the main analysis in multiple sensitivity analyses. Inverse probability weights were utilised as an alternative weighting scheme in place of overlap weights to estimate HRs. We also explored the use of the doubly robust approach, where covariates used to construct inverse probability weights were included in each model specification as explanatory variables. This approach was used to prevent model mis-specification in the generation of inverse probability weights or HRs in subsequent analyses. The risk of negative-outcome controls (any solid-organ malignancy) at 31-300 days from T_0 (test-date) was evaluated between SARS-CoV-2-infected cases and test-negatives; these outcomes were chosen as negative-outcome controls as no prior knowledge, to-date, supports the existence of a causal association between SARS-CoV-2-infection and cancer risk. Finally, given that individuals hospitalised for COVID-19 had increased risk of new-onset type-2 diabetes compared with test-negatives, to evaluate if diabetogenic risk was attributable to the physiological stress of hospitalization for respiratory-viral-infection or virus-specific factors, risk of diabetes post-COVID-19 hospitalization was contrasted against that following historical hospitalization for influenza from 1st Jan 2017-31st Dec 2022. Hospitalizations for influenza were already excluded from the COVID-19/test-negative cohorts earlier; the cohort of hospitalized influenza patients was identified using influenza-specific ICD-10 discharge codes ($J09^x$, $J10.0^x$, $J10.1^x$,$J10.8^x$) in national healthcare claims data from 1 January 2017 – 31 December 2022 , where ^{x} denotes that all other subcodes of the parent ICD-10 code were included.[2] The historical cohort of influenza hospitalizations (2017-2022) was not fully contemporaneous with COVID-19 hospitalisations from Sept 2021-Dec 2022, because imposition of public health measures in 2021 during the COVID-19 pandemic resulted in unprecedented drops in community transmission of influenza in Singapore.[3,4] Inclusion/exclusion criteria for construction of the historical influenza hospitalization cohort mirrored that of COVID-19 cases: T_0 (index-date) was taken as date-of-admission, and individuals aged<18 years, who died within 30 days of T_0, who were re-hospitalized for influenza or subsequently hospitalized for COVID-19 within 300 days of T_0, or who were previously diagnosed with type-2 diabetes/prediabetes as recorded in the NDD at any timepoint before T_0 were additionally excluded. Risk of post-acute type-2 diabetes 31-300 days following COVID-19 hospitalization was compared against the historical influenza hospitalization cohort (2017-2022), using the same analytical strategy of weighted competing-risk-regression, risk trajectories and excess burdens calculations as above, with influenza hospitalizations taken as the control group. A 95% confidence interval that excluded one was considered evidence of statistical significance. All analyses were conducted using R version 4.3.1.

© 2025 Wee LE et al. *JAMA Network Open*.

eFigure. Cohort Construction Flowchart

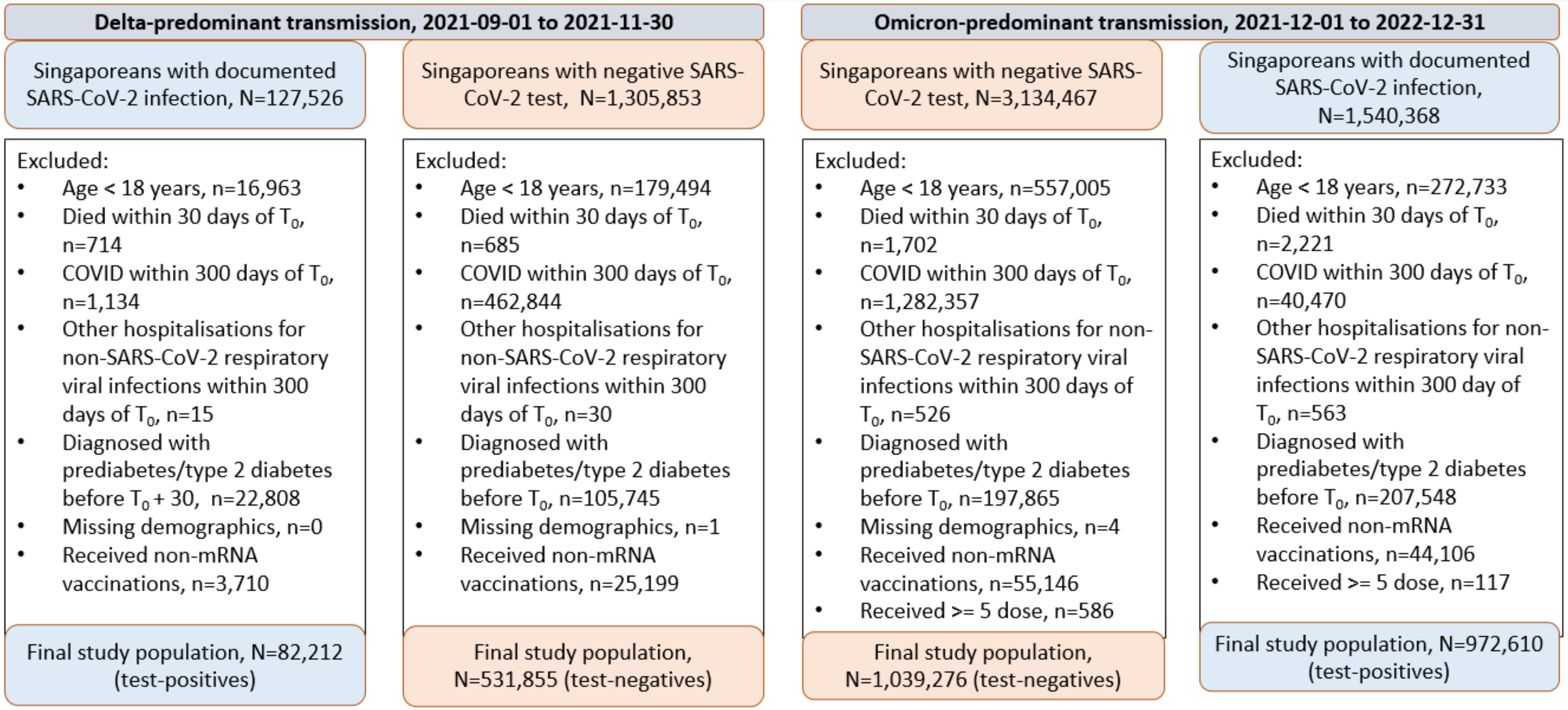

***Hospitalizations for non-SARS-CoV-2 respiratory viral infections included hospital admissions where the discharge diagnosis included influenza or respiratory-syncytial-virus (RSV); cases of influenza and RSV were identified using ICD-10 diagnosis codes in national healthcare claims data (influenza: J9-J10^{x}; RSV: J121, J205, J210, B974).[2] As physical stress following acute hospitalization for respiratory viral infection has been postulated to increase risk of new-onset diabetes in hospitalized patients through inducing stress hyperglycemia as a physiological response to severe illness,[5] individuals hospitalised for non-SARS-CoV-2 respiratory viral infection were additionally excluded from the cohort. Individuals vaccinated with non-mRNA vaccines or who received ≥5 vaccine doses were also additionally excluded, as these individuals formed a small non-representative minority of the study population.

© 2025 Wee LE et al. *JAMA Network Open*.

eReferences

1. Li F, Thomas LE, Li F. Addressing Extreme Propensity Scores via the Overlap Weights. Am J Epidemiol. 2019 Jan 1;188(1):250-257. doi: 10.1093/aje/kwy201.
2. Hamilton MA, Calzavara A, Emerson SD, Djebli M, Sundaram ME, Chan AK, Kustra R, Baral SD, Mishra S, Kwong JC. Validating International Classification of Disease 10th Revision algorithms for identifying influenza and respiratory syncytial virus hospitalizations. PLoS One. 2021 Jan 7;16(1):e0244746. doi: 10.1371/journal.pone.0244746.
3. Tan JY, Conceicao EP, Sim XYJ, Wee LEI, Aung MK, Venkatachalam I. Public health measures during COVID-19 pandemic reduced hospital admissions for community respiratory viral infections. J Hosp Infect. 2020 Oct;106(2):387-389. doi: 10.1016/j.jhin.2020.07.023
4. Chow A, Hein AA, Kyaw WM. Unintended Consequence: Influenza plunges with public health response to COVID-19 in Singapore. J Infect. 2020 Aug;81(2):e68-e69. doi: 10.1016/j.jinf.2020.04.035.
5. Rathmann W, Kuss O, Kostev K. Incidence of newly diagnosed diabetes after Covid-19. Diabetologia. 2022 Jun;65(6):949-954. doi: 10.1007/s00125-022-05670-0.

© 2025 Wee LE et al. *JAMA Network Open*.
