# Supplementary material for: Risk of New-Onset Type 2 Diabetes Among Vaccinated Adults After Omicron or Delta Variant SARS-CoV-2 Infection
Source: JAMA Netw Open. 2025 Apr 2;8(4):e252959. doi: 10.1001/jamanetworkopen.2025.2959 (PMC11966305; doi:10.1001/jamanetworkopen.2025.2959)
Supplement: Supplement 2. — Data Sharing Statement [file jamanetwopen-e252959-s002.pdf]

Data Sharing Statement

Wee. Risk of New-Onset Type 2 Diabetes Among Vaccinated Adults After Omicron or Delta Variant SARS-CoV-2 Infection. *JAMA Netw Open*. Published April 02, 2025. doi:10.1001/jamanetworkopen.2025.2959

Data
Data available: No

Additional Information
Explanation for why data not available: Individual data is not available due to personal data protection. Deanonymised datasets will be made available, subject to approval by the Ministry of Health, Singapore. All enquiries to be made to the corresponding author.
